# Beyond “Move More”: Combined Physical Activity and Sedentary Behavior Assessment in Individuals with MASLD from Southern Italy

**DOI:** 10.3390/jcm15062126

**Published:** 2026-03-11

**Authors:** Antonella Bianco, Claudia Beatrice Bagnato, Isabella Franco, Nicola Verrelli, Caterina Bonfiglio

**Affiliations:** 1 Laboratory of Movement and Wellness, National Institute of Gastroenterology IRCCS “Saverio de Bellis”, Castellana Grotte, 70013 Bari, Italyisabella.franco@irccsdebellis.it (I.F.); nicola.verrelli@irccsdebellis.it (N.V.); 2 Data Science, National Institute of Gastroenterology IRCCS “Saverio de Bellis”, Castellana Grotte, 70013 Bari, Italy; catia.bonfiglio@irccsdebellis.it

**Keywords:** metabolic dysfunction-associated steatotic liver disease, sedentary behavior, liver, actigraphy, physical activity, IPAQ

## Abstract

**Background**: In Southern Italy, metabolic dysfunction-associated fatty liver disease (MASLD) is rising despite adherence to traditional Mediterranean diets. Accurate assessment of physical activity (PA) and sedentary behavior is critical for effective non-pharmacological management but remains methodologically challenging. **Methods**: We compared subjective and objective PA measures in 133 adults (mean age 49.0 ± 9.8 years; BMI 35.7 ± 4.9 kg/m^2^) with moderate-to-severe MASLD. Participants completed the International Physical Activity Questionnaire–Short Form (IPAQ-SF) and wore an ActiGraph GT9X wrist accelerometer for seven days. **Results**: The IPAQ-SF significantly underestimated moderate PA by 865 min/week (*p* < 0.001) and reported 33.16 ± 14.78 min/week of vigorous activity not detected by accelerometry. Sedentary time was slightly underestimated (0.45 h/day, *p* = 0.05), with better overall agreement. Stratified analyses showed significant underestimation of sedentary behavior among women and participants <50 years. Spearman correlations were weak (rho = 0.14 for moderate PA; rho = 0.36 for sedentary behavior). Bland–Altman plots confirmed poor agreement for moderate PA but acceptable limits for sedentary estimates. **Conclusions**: In high-risk Southern Italian populations with MASLD, reliance on self-reported PA may lead to inaccurate clinical guidance. Integrating objective monitoring with subjective tools is essential to deliver precise, individualized exercise prescriptions beyond generic “move more” recommendations.

## 1. Introduction

In 2023, based on a greater understanding of the disease and in accordance with EASL-EASD-EASO Clinical Practice Guidelines, non-alcoholic fatty liver disease (NAFLD) was officially renamed metabolic dysfunction-associated steatotic liver disease (MASLD) [1]. This terminology more accurately reflects the metabolic underpinnings of the disease, moving beyond the previous definition, which was based only on the presence of hepatic steatosis in the absence of significant alcohol consumption [2]. In contrast, MASLD is defined by the presence of hepatic steatosis in conjunction with at least one key cardiometabolic risk factor, including obesity, type 2 diabetes, dyslipidemia, hypertension, and insulin resistance [3]. MASLD is now recognized as a leading hepatic manifestation worldwide, with a global prevalence of approximately 30% among adults and approximately 20% in Italy [4,5]. Regional estimates suggest an even higher disease burden in southern Italy, where, despite its historical link to the Mediterranean diet, traditionally associated with a lower metabolic risk, high rates of obesity, poor adherence to physical activity guidelines, and a marked tendency toward sedentary behaviour (SB) coexist. This so-called “Mediterranean paradox” reflects a deep transition in lifestyle [6], driven by modern socio-economic factors including rapid urbanization, a shift towards sedentary occupations, and increased availability of processed foods, despite the historical link to the Mediterranean diet.

The impact of MASLD extends well beyond the clinical domain. In Italy, the disease imposes a substantial economic burden on the National Health Service, with costs projected to rise as the disease progresses [5]. Indeed, if not adequately managed, MASLD can progress to more severe forms, such as metabolic dysfunction-associated steatohepatitis (MASH), and, in advanced cases, may lead to the development of hepatocellular carcinoma [1]. The global rise in this condition is closely linked to the ongoing obesity pandemic, as approximately 75% of individuals with obesity are affected by MASLD [7]. However, it is not body weight alone that determines risk; lifestyle also plays a critical role. It is now well established that prolonged SB constitutes an independent risk factor for the development and progression of MASLD [8,9], even after accounting for the overall level of physical activity [10]. This association is supported by multisystemic alterations induced by muscle inactivity, such as reduced lipoprotein lipase (LPL) activity and increased skeletal insulin resistance; these mechanisms promote the transfer of lipids to the liver, increasing oxidative stress and promoting a pro-inflammatory state that favors the progression of steatosis [11,12].

In particular, among individuals with obesity, those who are sedentary exhibit a significantly higher risk of hepatic steatosis compared to their similarly weighted but more physically active counterparts [13]. In the absence of approved pharmacological therapies for MASLD, lifestyle intervention, particularly increasing physical activity, represents one of the non-pharmacological cornerstone strategies for managing the disease [1,14].

Numerous studies demonstrate that regular physical activity confers significant benefits in individuals with MASLD, improving insulin sensitivity, body composition, and liver-related parameters [15]. Nevertheless, it is well documented that these individuals tend to engage in lower levels of physical activity compared with the general healthy population [14]. This reinforces a vicious cycle linking SB, metabolic dysfunction, and liver injury.

In an era in which precision medicine is redefining the management of metabolic liver diseases, physical activity should no longer be considered merely a general lifestyle recommendation but a genuine therapeutic tool in the context of MASLD. However, its clinical and predictive potential remains largely untapped as long as we continue to treat physical activity as a homogeneous and undifferentiated construct. An accurate assessment of not only quantity but also intensity, type, and concomitant SB is now essential. This assessment provides the basis for identifying individual predictive parameters, which are fundamental for designing increasingly personalized interventions, correctly interpreting study results, and ultimately maximizing therapeutic benefits in patients with MASLD [16,17].

In recent years, several methods have been developed to assess physical activity and SB. These approaches can be broadly categorized into two groups: subjective and objective measurements.

Self-reported assessments, typically administered via questionnaires, are among the most widely used tools for evaluating both physical activity and SB, owing to their low cost and ease of implementation. Through a series of structured questions, they capture information on both behavioral domains [18]. These instruments are particularly valuable in large-scale epidemiological studies; however, they are subject to several limitations, including recall and social desirability bias, and difficulty accurately quantifying the full spectrum of daily physical activity and SB [19,20]. Although self-reported questionnaires are practical and widely used, more accurate and objective assessments often rely on electronic devices such as calorimeters, pedometers, and accelerometers [19]. Accelerometers, in particular, are validated electronic devices that provide detailed, objective data on the frequency, duration, intensity, and type of physical activity, as well as the amount of time spent in SB [19]. However, limited data are available that compare subjective and objective measures of physical activity and SB in patients with MASLD, particularly in the population of southern Italy.

Given the high prevalence of SB and metabolic risk in southern Italy, we hypothesized that in patients with MASLD, we would observe: (1) poor agreement between measures of moderate–vigorous physical activity (MVPA) obtained using IPAQ-SF and ActiGraph, with significant underestimation by the subjective instrument; and (2) significant differences in self-assessment accuracy based on age and gender. Consequently, only the integration of objective and subjective analyses can provide a clinically useful picture of motor behaviour.

To address this gap, our study aims to characterize physical activity levels and SB in a cohort of adults with MASLD from Southern Italy, using both subjective and objective assessment tools.

## 2. Materials and Methods

### 2.1. Participants and Study Design

Participants were recruited between October 2023 and October 2025 at the Movement and Wellness Laboratory of IRCCS “Saverio de Bellis” (Castellana Grotte, Italy) through referrals from general practitioners or voluntary responses to an online questionnaire disseminated via the institution’s website and social media. This cross-sectional study represents the baseline assessment of a randomized controlled trial (ClinicalTrials.gov ID: NCT06186869) evaluating the effects of the Mediterranean diet combined with two exercise regimens—differing in type and intensity—on systemic inflammation and related biomarkers. A total of 133 adults diagnosed with MASLD provided written informed consent. The study was conducted in accordance with the Declaration of Helsinki and approved by the local Ethics Committee (Protocol No. 1253/CE, 7 June 2023). Inclusion and exclusion criteria have been described in detail elsewhere [4].

In accordance with the international definition of MASLD, daily alcohol consumption of less than 30 g for men and 20 g for women was required [1].

### 2.2. Data Collection

Weight and height were measured using SECA instruments (model 700 and model 206; SECA, Hamburg, Germany), and standard methods were employed to perform biochemical measurements. Each participant was provided with a medical history questionnaire to gather information on their clinical history, the presence or absence of diseases, allergies, medication use, and other risk factors (such as smoking, alcohol consumption, stress, and sleep patterns), as well as educational qualifications and occupation.

Physical activity levels and SB were assessed subjectively using a validated questionnaire (IPAQ-SF) [21] and objectively using an accelerometer (ActiGraph GT9X, ActiGraph Corporation, Pensacola, FL, USA) [22].

### 2.3. Physical Activity and Sedentary Behaviour Assessment

#### 2.3.1. International Physical Activity Questionnaire - Short Form (IPAQ-SF)

Self-reported physical activity was assessed using the short form of the International Physical Activity Questionnaire (IPAQ-SF), a validated tool widely used in adults aged 18–65 [18,19,20,21]. Administered via face-to-face interview, the IPAQ-SF quantifies time spent in vigorous, moderate, and walking activities, as well as SB on weekdays and weekend days. Participants were provided with examples to aid in classifying activity intensity. Total physical activity was calculated by multiplying minutes per session by frequency (days/week) for each intensity level, while sedentary time was expressed in hours per weekday and weekend day.

#### 2.3.2. Accelerometer–ActiGraph

Physical activity and SB were objectively measured using the ActiGraph GT9X accelerometer, a widely used wearable device equipped with a triaxial accelerometer, gyroscope, and magnetometer [22]. Participants wore the device on their dominant wrist for seven consecutive days, removing it only during water exposure. Monitoring days with ≥14 h of wear time between 7 a.m. and 10 p.m. were considered valid; Data from the first and last days were excluded a priori to minimise potential distortions due to reactivity and artefacts related to device insertion/removal, in line with established accelerometric methodology [23,24].

Participants were included in the analysis if they recorded ≥4 weekdays and ≥2 weekend days, meeting this criterion.

Data were collected at 30 Hz and processed in 60-s epochs using ActiLife software (v6.14.0). Activity intensities were classified using Troiano’s 2008 [25] cut-offs for adults: SB (0–199 counts/min), light (LPA: 200–2019), moderate (MPA: 2020–5998), and vigorous (VPA: ≥5999); MVPA combined MPA and VPA. Daily time spent in each intensity was expressed in minutes, while SB was converted to hours per day.

Sleep periods were excluded from sedentary behavior estimates. Sleep onset and offset were identified using participant-completed wear-time diaries, supplemented by visual inspection of activity profiles to confirm prolonged bouts of inactivity.

We chose wrist placement for several reasons: (1) greater comfort and acceptability by participants, especially in prolonged monitoring protocols; (2) recent evidence supporting the use of the wrist in epidemiological studies to improve adherence [26,27]; (3) the need to minimise discomfort and interference with participants’ daily lives, considering their specific clinical conditions.

To ensure temporal alignment, the IPAQ-SF was administered immediately after the 7-day monitoring period, with explicit instructions for participants to recall physical activity and SB performed during the same week of accelerometer wear.

### 2.4. Statistical Analyses

All analyses were performed using Stata 19 (StataCorp LLC, College Station, TX, USA), with statistical significance set at *p* ≤ 0.05. Normality and homogeneity of variances were assessed using the Shapiro–Wilk and Levene tests. If *p*-value > 0.05, the normality assumption is satisfied and the data are considered normally distributed. If *p* < 0.05, the data deviate significantly from normality, justifying the use of non-parametric tests. As MPA, MVPA, and SB showed non-normal distributions, group comparisons between IPAQ-SF and ActiGraph data (overall, by sex, and by age <50 vs. ≥50 years) were conducted using the Wilcoxon signed-rank test. Results are reported as means with standard error (SEM) or standard deviation (SD), as appropriate. Effect sizes were measured using Cohen’s d for paired data, calculated by dividing the mean of the differences by the standard deviation of the differences. Cohen’s d conventional effect size cutoffs [small (0.2), medium (0.5), and large (0.8)].

The MedCalc 23.2.0 program was used to calculate the size using the method described by Lu et al. [28].

Spearman’s rank correlation coefficients were calculated to evaluate the intensity and direction between IPAQ-SF and ActiGraph estimates, with correlations interpreted as: weak (0–0.39), moderate (0.40–0.69), or strong (>0.70). Correlations for MPA and SB were visualized using scatter plots with superimposed linear trends.

Agreement between methods was evaluated via Bland–Altman analysis [29,30], plotting the mean of the two measurements (*x*-axis) against their difference (*y*-axis). The plot displays bias (mean difference), limits of agreement (bias ± 1.96 SD), and 95% confidence intervals. Random scatter around the bias line indicates good agreement, whereas systematic deviations or points beyond the limits suggest methodological discrepancies.

## 3. Results

One hundred and thirty-three adults with moderate-to-severe MASLD (mean age: 49.0 ± 9.8 years; BMI: 35.7 ± 4.9 kg/m^2^; CAP: 304.6 ± 35.3 dB/m) were included in the study. Nearly half were women (48.9%), and 58.7% reported sedentary occupations (Table 1).

### 3.1. Comparisons Between the ActiGraph and IPAQ-SF Data

Objective (ActiGraph) and subjective (IPAQ-SF) assessments revealed significant discrepancies in physical activity estimates (Table 2).

The IPAQ-SF markedly underestimated moderate physical activity (MPA) by 865 min/week (*p* < 0.001) and combined moderate-to-vigorous physical activity (MVPA) by 840 min/week (*p* < 0.001). Conversely, it reported 33.2 min/week of vigorous activity (VPA), which accelerometry did not detect (*p* < 0.001). For SB, the IPAQ-SF slightly underestimated total weekly SB by 0.45 h/day (*p* = 0.05), with a more pronounced underestimation on weekdays (0.79 h/day, *p* = 0.005); weekend SB estimates were comparable (*p* = 0.97).

### 3.2. Associations Between Data from the ActiGraph and IPAQ-SF

**Figure 1:**
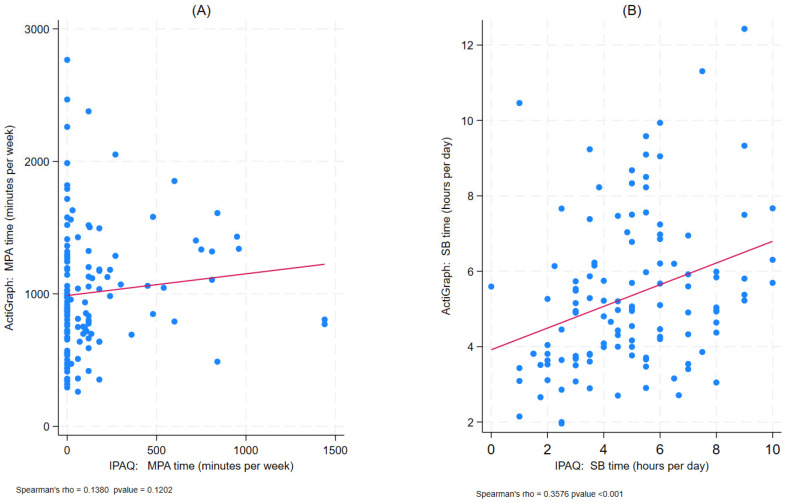
Correlations between the IPAQ-SF and ActiGraph (Spearman correlation) measures in MASLD patients: for MPA—Panel (**A**); for SB—Panel (**B**). rho = Spearman’s Correlation Coefficient; MPA = Moderate Physical Activity; SB: Sedentary Behaviour; IPAQ-SF: International Physical Activity Questionnaire–Short Form.

Spearman correlations between methods were weak, where rho = 0.14 for MPA (*p* = 0.12) and rho = 0.36 for SB (*p* < 0.001) (Figure 1).

**Figure 2:**
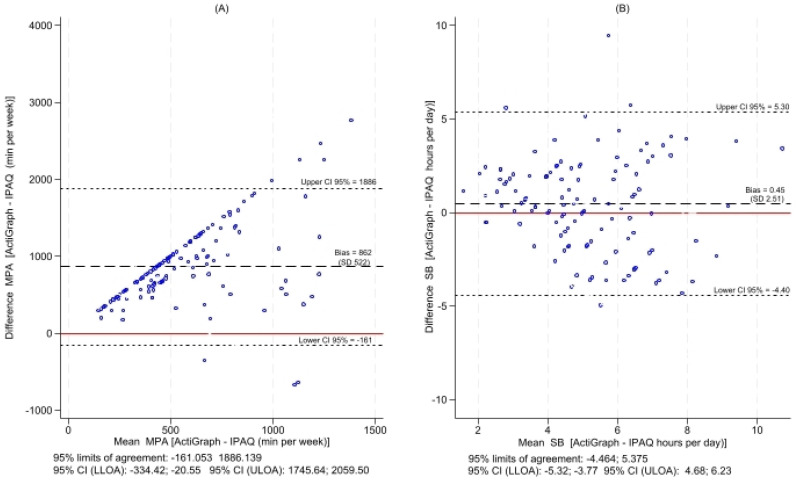
Bland–Altman graphs of MASLD patients with measurements obtained by IPAQ-SF and ActiGraph. The *x*-axis (mean of the measurements obtained by IPAQ-SF and ActiGraph) and the *y*-axis (difference in the measurements obtained by IPAQ-SF and ActiGraph). Panel (**A**) = MPA; Panel (**B**) = SB. MPA = Moderate Physical Activity; SB: Sedentary Behaviour; IPAQ-SF: International Physical Activity Questionnaire–Short Form. The red line indicates the maximum agreement between the two methods.

Bland–Altman plots confirmed poor agreement for MPA (bias: +862 min/week; wide limits of agreement: 95% limits of agreement: −161.05; 1886.14) but acceptable agreement for SB (bias: +0.45 h/day and 95% limits of agreement: −4.46; 5.37) (Figure 2).

### 3.3. Comparisons Between the ActiGraph and IPAQ-SF Data by Age and Sex

Stratified analyses showed that SB underestimation was significant only in participants aged <50 years and in women. In participants <50 years, SB was underestimated by 1.11 h/day on weekdays and 0.78 h/day overall (*p* ≤ 0.01), while no significant differences were observed in those ≥50 years (Table 3).

Figure S1 displays the correlations between IPAQ-SF and ActiGraph data, while Figure S2 displays the Bland–Altman graphs for MPA and SB in MASLD subjects, stratified by age (<50 vs. ≥50 years).

Among women, IPAQ-SF underestimated weekday SB by 0.94 h/day and total SB by 0.82 h/day (*p* = 0.005 for both) (Table 4).

Figure S3 displays the correlations between IPAQ-SF and ActiGraph data, while Figure S4 displays the Bland–Altman graphs for MPA and SB in MASLD subjects, stratified by sex.

MPA and MVPA remained substantially underestimated across all subgroups (*p* < 0.001), with the largest gaps seen in women (MPA difference: 1067 min/week) and younger adults (MPA difference: 934 min/week).

VPA was consistently reported via IPAQ-SF but never recorded by accelerometry, with significant discrepancies in both sexes and age groups (*p* < 0.01). LPA, captured only by accelerometry, averaged > 2900 min/week, highlighting high volumes of low-intensity movement not reflected in self-reports.

## 4. Discussion

Physical activity is a fundamental pillar in non-pharmacological approaches to the treatment and management of MASLD; its accurate assessment, therefore, plays an important role in both clinical and research settings to optimize the effectiveness of therapeutic interventions.

Our results show limited agreement between the two assessment methods considered in terms of time spent in MVPA, while showing a good level of agreement in the estimation of sedentary time.

In particular, the accelerometer recorded a significantly higher total volume of physical activity than the IPAQ-SF. The latter underestimated the time spent in MPA and overestimated the time spent in VPA, while providing comparable estimates for SB.

A particularly relevant finding is that, although some participants stated in the questionnaire that they engaged in VPA, no episodes of such intensity were detected by the accelerometer. This discrepancy can be attributed to two sets of factors.

On the one hand, we can assume that some participants overestimated the intensity of the exercise they performed, perceiving as “vigorous” an activity that, in objective terms, was not. Several studies have shown that individuals with MASLD perceive greater effort at similar exercise thresholds compared to healthy individuals [14,31]. This discrepancy could be attributable to individual factors such as low physical fitness, a lack of awareness of the intensity of effort, or differences in perceived effort [32]. In a cohort characterised by class II obesity (mean BMI 35 kg/m^2^), prolonged engagement in metabolically intense activities is physiologically and mechanically unlikely. Participants likely confused the increased perceived exertion, resulting from the reduced cardiorespiratory efficiency typical of the condition, with intense metabolic activity. This misalignment between relative (perceived) and absolute (metabolic) intensity highlights the limitation of subjective tools alone in complex clinical populations. On the other hand, it should be noted that, as reported in the literature, ActiGraph may underestimate energy expenditure both during low-intensity activities (e.g., slow walking or standing) and during high-intensity exercise, as acceleration fluctuations may be too slight or too rapid to be accurately detected by the sensor [33,34]. Consequently, integrating objective and subjective monitoring is essential to differentiate between metabolic intensity and perceived exertion, ensuring safe and effective exercise prescriptions despite technological limitations.

The high volume of LPA recorded by accelerometry also requires cautious interpretation: although it may reflect actual incidental movement (e.g., slow walking, light housework), wrist-worn devices are susceptible to capturing arm movements that do not correspond to whole-body energy expenditure. It is crucial to recognize that accelerometers quantify mechanical movement (acceleration) rather than direct metabolic energy expenditure. Consequently, high-frequency wrist movements (e.g., gesturing, handwashing) may generate activity counts without corresponding whole-body energy costs, potentially leading to intensity misclassification, particularly when using cut-offs validated for hip placement. This methodological consideration is particularly relevant in clinical populations with obesity, where movement patterns may differ significantly from those of healthy adults [26]. Although placement on the wrist, compared to other body locations, makes the device more susceptible to non-locomotor movements (e.g., hand gestures or passive oscillations), it is widely used in population studies [26] due to the greater comfort and compliance associated with it [27].

This discrepancy is consistent with a recent systematic review, which highlights how self-administered questionnaires often underestimate physical activity, while accelerometers, thanks to continuous recording, capture overlooked or forgotten aspects [20]. Furthermore, the substantial underestimation of MPA by the IPAQ-SF reflects a combination of perceptual, methodological, and psychosocial factors. In patients with MASLD and obesity, altered perception of effort may lead to classifying low-intensity activities as “moderate”. This bias, combined with the limitations of wrist accelerometry (sensitive to non-locomotor movements) and social desirability, may explain the observed discrepancy. These mechanisms underscore the need to integrate objective monitoring and contextualised interpretation in the assessment of physical activity in clinical populations with MASLD.

Unlike physical activity, agreement for SB was good, with low bias and acceptable limits of agreement in Bland–Altman analyses. This suggests that participants more easily recognise SB, as they are associated with daily routines such as work, time spent watching television, and time spent eating meals [35]. The differential underestimation of SB on weekdays compared to weekends may reflect differences in temporal structure and accuracy of recall. Weekdays, characterised by more predictable routines (e.g., work schedules, fixed meal times), may be easier to summarise in questionnaires, but also more susceptible to social desirability bias, leading participants to underestimate time spent sitting. In contrast, the greater variability of weekend activities may result in less systematic reporting errors.

The stratified analysis by gender and age revealed a particularly interesting aspect: among women and individuals under the age of 50, there was a statistically significant difference between self-reported sedentary hours and those measured objectively. This discrepancy between genders and ages could be attributable to several mechanisms. Firstly, social desirability and self-presentation bias could lead people, particularly women, to report less sedentary time. Women tend to be more sensitive to social evaluation and to report healthier and more socially desirable behaviors [36,37]. Furthermore, for women, we hypothesize that the multitasking typical of domestic and work responsibilities may make it more difficult to segment and remember sedentary time. Secondly, in younger individuals (<50 years), we assume that the underestimation could stem from a more active perception of oneself or greater confidence in one’s physical condition. In this age group, physical activity is usually overestimated, while sedentary time is not perceived as risky behaviour [38,39].

Current recommendations on physical activity are derived from guidelines formulated for the general population and do not include specific parameters for MASLD: they recommend 75–150 min of vigorous activity or 150–300 min of moderate activity per week [40]. However, physical activity alone, even if it complies with these recommendations, may not be sufficient to achieve significant metabolic and hepatic benefits. In this context, it is very important to explain the distinction between generalised physical activity and exercise: while physical activity includes any bodily movement that increases energy expenditure, exercise is defined as a planned, structured, and repetitive activity with the specific aim of improving health and fitness [41].

It is now well established in the literature that different types of structured physical exercise, whether aerobic, resistance, or combined, can reduce liver fat content in patients with MASLD, regardless of weight loss [42,43,44,45].

However, despite the evidence of the benefits of physical exercise, patients with MASLD rarely achieve sufficient levels of intensity to obtain these effects, highlighting the urgent need to transition from generic recommendations to individualized prescriptions based on each patient’s metabolic, functional, and behavioral profiles.

This becomes even more important in the context of southern Italy, where national surveillance data on obesity rates, sedentary lifestyles, and low levels of physical activity are much higher than in the rest of the country (72.5% vs. 57.1%, respectively) [41]. This gap probably reflects a complex intertwining of socio-economic, environmental, and cultural factors, including limited access to adapted physical exercise facilities and an organization that is less conducive to active travel. This study partially fills the critical gap in the literature on MASLD in subjects in southern Italy. However, some limitations should be acknowledged. (1) The use of the ActiGraph, worn on the wrist, may be subject to too much oscillation, leading to an overestimation of the total volume of physical activity (for non-locomotor movements) but an underestimation of intensity. However, it should be noted that the choice of placement is not accidental. The ActiGraph GT9X was worn on the wrist to optimise comfort and compliance among participants during the 7-day monitoring period, in line with recent population-based studies [26,27]. Although hip placement remains the traditional reference for locomotor activity, wrist-worn accelerometry offers practical advantages in clinical cohorts, albeit with recognized trade-offs in intensity classification accuracy. Additionally, accelerometers measure movement acceleration rather than direct energy expenditure, which, combined with wrist placement and hip-derived cut-offs, can contribute to intensity misclassification. (2) The use of Troiano et al. [25] cut-offs for wrist data is a limitation, as these thresholds have been validated for the hip, and the different wearing locations may alter the classification of intensities [46]. The choice was dictated by the options available in the analysis software (Actilife v6.14.0), which at the time of analysis only supported the Troiano and Freedson algorithms. While recognising that this procedure may influence the absolute estimate of physical activity volumes, our conclusions are based primarily on relative comparisons between groups/conditions within the cohort, an approach that reduces the impact of potential bias on comparative results. Future studies will benefit from the adoption of wrist-specific algorithms and raw data processing to improve the accuracy of estimates and the generalisability of results. (3) The IPAQ-SF, due to the limited number of questions, does not allow for a comprehensive understanding of the different types of movement and daily activities performed by participants. Furthermore, although adequate for methodological analysis, the sample size may not be sufficient to generalise the results to larger or more diverse populations.

However, the strengths are equally significant: the sample is well characterised clinically, the administration of the IPAQ-SF at the end of the monitoring week ensures temporal comparability, and the sample size is consistent for a methodological study on MASLD.

In light of the discrepancies highlighted by the study, we propose a multi-tiered clinical workflow to optimise assessment accuracy while managing resources. Subjective questionnaires can serve as an initial screening tool for general activity patterns, given their acceptability for sedentary behaviour. However, for high-risk patients (e.g., severe obesity, MASLD), objective monitoring should supplement self-assessments to ensure accurate baseline profiling for personalised prescriptions and to subsequently monitor intervention outcomes. This integrated approach facilitates the transition from generic recommendations to dose-specific prescriptions, ensuring that interventions are both safe and metabolically effective.

In future studies, it will be appropriate to increase the sample size to examine a greater number of variables and a more heterogeneous population. In addition, consideration could be given to wearing the ActiGraph on other parts of the body, such as the waist, to reduce the frequent fluctuations caused by positioning on the wrist. Finally, in addition to using questionnaires and accelerometers to assess physical activity, it would be useful to measure the physical capacity of the subjects in order to obtain a more complete picture of their characteristics.

## 5. Conclusions

Physical activity remains a pillar in the management of MASLD, but generic recommendations to “move more” are often insufficient. Unlike previous studies conducted on general populations, our work provides an assessment of motor behaviour in a specific cohort of MASLD patients residing in Southern Italy, a region with a high metabolic risk. The discrepancies observed between subjective and objective measures highlight that relying solely on self-reported data can lead to inaccurate clinical indications regarding activity intensity. In this context, the integration of objective monitoring with subjective tools is necessary for a comprehensive assessment of physical activity patterns. Although spontaneous movement is beneficial, accurate profiling is important for the design of personalised, dosed and supervised interventions. Ultimately, accurate assessment is a prerequisite for translating physical activity into an effective therapeutic tool for MASLD, going beyond generic recommendations.

## Figures and Tables

**Table 1:** Characteristics of the sample.

Variables	All Sample
N	133
Age (yrs)	49.03 (9.75)
Sex (%)	
Female	65 (48.9)
Male	68 (51.1)
BMI (kg/m^2^)	35.74 (4.88)
Weight (kg)	99.97 (15.84)
CAP (dB/m)	304.59 (35.30)
Smoker (%)	
Never/Former	113 (84.96)
Current	20 (15.04)
Marital Status (%)	
Single	33 (24.81)
Married or living together	91 (68.42)
Separated or Divorced	7 (5.26
Widow/er	2 (1.50)
Education (%)	
Primary school	2 (1.50)
Secondary school	19 (14.29)
High School	76 (57.14)
Graduate	36 (27.07)
Job (%)	
Unemployed	17 (12.78)
Farmer	4 (3.01)
Worker	9 (6.77)
Clerk	57 (42.86)
Merchant	7 (5.26)
Freelancer	22 (16.54)
Craftsman	3 (2.26)
Housewife	7 (5.26)
Retired	7 (5.26)
Type of job (%)	
Very tiring	14 (10.53)
Tiring	19 (14.29)
Sedentary	78 (58.65)
Unclassified	22 (16.54)

Age, BMI, weight, and CAP are expressed as Mean and Standard Deviation. The other data are expressed as a percentage (%). BMI: Body Mass Index; CAP: Controlled Attenuation Parameter.

**Table 2:** Comparison (Wilcoxon Signed-Rank Test t) of the measurements obtained by IPAQ-SF (walking, MPA, VPA, MVPA, and SB) and ActiGraph (LPA, MPA, VPA, MVPA, and SB) of patients with MASLD.

Variables	ActiGraph	IPAQ-SF	Difference	Cohen’s d	*p*-Value ^¥^
	Mean (SD)	Mean (SD)	Mean (SEM)		
Walking (min per week)	-	295.86 (359.59)	-		-
LPA (min per week)	2903.23 (471.02)	-	-		-
MPA (min per week)	1015.75 (470.58)	150.05 (272.06)	865.11 (46.45)	1.65	<0.001
VPA (min per week)	0.00 (0.00)	33.16 (170.43)	−33.16 (14.78)	0.20	<0.001
MVPA (min per week)	1015.75 (470.58)	175.30 (326.31)	840.45 (50.47)	1.46	<0.001
SB 4 weekdays (hours/day)	5.98 (2.17)	5.19 (2.56)	0.79 (0.25)	0.28	0.005
SB weekend (hours/day)	4.54 (2.06)	4.57 (2.64)	−0.025 (0.28)	0.01	0.9701
SB total week (hours/day)	5.32 (2.00)	4.86 (2.23)	0.45 (0.23)	0.18	0.0501

^¥^ Wilcoxon signed-rank test, LPA = Light Physical Activity, MPA = Moderate Physical Activity, VPA = Vigorous Physical Activity, MVPA = Moderate and Vigorous Physical Activity; SB = Sedentary Behaviour.

**Table 3:** Comparison (Wilcoxon signed-rank test) of the measurements obtained by IPAQ-SF (walking, MPA, VPA, MVPA, and SB) and ActiGraph (LPA, MPA, VPA, MVPA, and SB) of patients with MASLD by age (<50 vs. ≥50 years).

Compared Measurements	ActiGraph	IPAQ-SF	Difference	*p*-Value ^¥^
	Mean (SD)	Mean (SD)	Mean (SEM)	
<50 years				
Walking (min per week)	-	309.57 (349.12)	-	
LPA (min per week)	3478.52 (552.55)	-	-	
MPA (min per week)	1070.31 (554.55)	136.425 (226.69)	933.89 (74.49)	<0.001
VPA (min per week)	0.00 (0.00)	12.29 (41.53)	−12.29 (5.32)	0.0078
MVPA (min per week)	1070.31 (554.55)	148.92 (224.69)	921.39 (75.07)	<0.001
SB 4 weekdays (hours/day)	6.07 (2.35)	4.96 (2.56)	1.11 (0.31)	0.0013
SB weekend (hours/day)	4.77 (2.60)	4.34 (2.53)	0.43 (0.41)	0.4365
SB total week (hours/day)	5.44 (2.38)	4.66 (2.19)	0.78 (0.29)	0.0098
≥50 years				
Walking (min per week)	-	278.41 (265.44)	-	
LPA (min per week)	3606.47 (568.80)	-	-	
MPA (min per week)	960.56 (378.45)	160.97 (316.17)	799.59 (56.26)	<0.001
VPA (min per week)	0.00 (0.00)	53.04 (232.43)	−53.04 (27.98)	<0.001
MVPA (min per week)	960.56 (378.45)	214.79 (401.09)	745.76 (66.06)	<0.001
SB 4 weekdays (hours/day)	5.90 (2.04)	5.38 (2.56)	0.53 (0.38)	0.2794
SB weekend (hours/day)	4.35 (1.56)	4.77 (2.79)	−0.42 (0.39)	0.4364
SB total week (hours/day)	5.21 (1.69)	5.03 (2.32)	0.17 (0.34)	0.7739

^¥^ Wilcoxon signed-rank test, LPA = Light Physical Activity, MPA = Moderate Physical Activity, VPA = Vigorous Physical Activity, MVPA = Moderate and Vigorous Physical Activity, SB = Sedentary Behaviour.

**Table 4:** Comparison (Wilcoxon Signed-Rank Test) of the measurements obtained by IPAQ-SF (walking, MPA, VPA, MVPA, and SB) and ActiGraph (LPA, MPA, VPA, MVPA, and SB) of patients with MASLD by sex.

Compared Measurements	ActiGraph	IPAQ-SF	Difference	*p*-Value ^¥^
	Mean (SD)	Mean (SD)	Mean (SEM)	
Female				
Walking (min per week)	-	258.95 (275.07)	-	
LPA (min per week)	3336.88 (564.48)	-	-	
MPA (min per week)	1225.03 (502.50)	158.13 (249.86)	1066.90 (69.62)	<0.001
VPA (min per week)	0.00 (0.00)	10.63 (31.26)	−10.63 (3.93)	0.0078
MVPA (min per week)	1225.03 (502.50)	168.93 (251.94)	1056.10 (69.87)	<0.001
SB 4 weekdays (hours/day)	5.78 (2.26)	4.84 (2.36)	0.94 (0.35)	0.005
SB weekend (hours/day)	4.38 (2.14)	3.96 (2.16)	0.42 (0.36)	0.3627
SB total week (hours/day)	5.16 (2.10)	4.35 (1.99)	0.82 (0.30)	0.005
Male				
Walking (min per week)	-	329.03 (338.62)	-	
LPA (min per week)	3766.85 (471.60)	-	-	
MPA (min per week)	811.89 (334.32)	141.32 (300.72)	670.57 (51.28)	<0.001
VPA (min per week)	0.00 (0.00)	55.82 (236.90)	−55.82 (28.94)	<0.001
MVPA (min per week)	811.89 (334.32)	197.99 (393.21)	613.90 (61.26)	<0.001
SB 4 weekdays (hours/day)	6.18 (2.11)	5.53 (2.72)	0.65 (0.37)	0.1906
SB weekend (hours/day)	4.70 (2.07)	5.15 (2.98)	−0.45 (0.43)	0.3692
SB total week (hours/day)	5.46 (1.97)	5.35 (2.41)	0.11 (0.33)	0.8224

^¥^ Wilcoxon signed-rank test, LPA = Light Physical Activity, MPA = Moderate Physical Activity, VPA = Vigorous Physical Activity, MVPA = Moderate and Vigorous Physical Activity, SB = Sedentary Behaviour.

## Data Availability

The original data presented in the study are openly available in FigShare at https://doi.org/10.6084/m9.figshare.30772772.

## Supplementary Materials

The following supporting information can be downloaded at: https://www.mdpi.com/article/10.3390/jcm15062126/s1, Correlations between IPAQ-SF and ActiGraph data stratified by age (<50 vs. ≥50 years)_Figure S1, Bland–Altman graphs for MPA and SB in MASLD subjects, stratified by age (<50 vs. ≥50 years)_Figure S2, Correlations between IPAQ-SF and ActiGraph data stratified by sex_Figure S3, Bland–Altman graphs for MPA and SB in MASLD subjects, stratified by sex_Figure S4.

## Abbreviations

The following abbreviations are used in this manuscript:

BMI	Body Mass Index
CAP	Controlled Attenuation Parameter
IPAQ-SF	International Physical Activity Questionnaire–Short Form
LPA	Light Physical Activity
MASLD	Metabolic Dysfunction-Associated Steatotic Liver Disease
MPA	Moderate Physical Activity
MVPA	Moderate-to-Vigorous Physical Activity
SB	Sedentary Behaviour
VPA	Vigorous Physical Activity

## References

[1] (2024). EASL-EASD-EASO Clinical Practice Guidelines on the Management of Metabolic Dysfunction-Associated Steatotic Liver Disease (MASLD). Obes. Facts.

[2] Rinella M.E. (2015). Nonalcoholic fatty liver disease: A systematic review. JAMA.

[3] Yanai H., Adachi H., Hakoshima M., Iida S., Katsuyama H. (2023). Metabolic-Dysfunction-Associated Steatotic Liver Disease-Its Pathophysiology, Association with Atherosclerosis and Cardiovascular Disease, and Treatments. Int. J. Mol. Sci..

[4] Bagnato C.B., Bianco A., Bonfiglio C., Franco I., Verrelli N., Carella N., Shahini E., Zappimbulso M., Giannuzzi V., Pesole P.L. (2024). Healthy Lifestyle Changes Improve Cortisol Levels and Liver Steatosis in MASLD Patients: Results from a Randomized Clinical Trial. Nutrients.

[5] Torre E., Di Matteo S., Martinotti C., Bruno G.M., Goglia U., Testino G., Rebora A., Bottaro L.C., Colombo G.L. (2024). Economic Impact of Metabolic Dysfunction-Associated Steatotic Liver Disease (MASLD) in Italy. Analysis and Perspectives. Clin. Outcomes Res. CEOR.

[6] Luconi E., Tosi M., Boracchi P., Colonna I., Rappocciolo E., Ferraretto A., Lorenzini E.C. (2024). Italian and Middle Eastern adherence to Mediterranean diet in relation to Body Mass Index and non-communicable diseases: Nutritional adequacy of simulated weekly food plans. J. Transl. Med..

[7] Quek J., Chan K.E., Wong Z.Y., Tan C., Tan B., Lim W.H., Tan D.J.H., Tang A.S.P., Tay P., Xiao J. (2023). Global prevalence of non-alcoholic fatty liver disease and non-alcoholic steatohepatitis in the overweight and obese population: A systematic review and meta-analysis. Lancet. Gastroenterol. Hepatol..

[8] Hallsworth K., Thoma C., Moore S., Ploetz T., Anstee Q.M., Taylor R., Day C.P., Trenell M.I. (2015). Non-alcoholic fatty liver disease is associated with higher levels of objectively measured sedentary behaviour and lower levels of physical activity than matched healthy controls. Frontline Gastroenterol..

[9] Wang J., Zhao J., Zhong Y., He C., Hu F. (2025). Healthy Lifestyle and Metabolic Dysfunction-Associated Steatotic Liver Disease: A Study of the Efficacy of Fatty Liver Regression. Clin. Transl. Gastroenterol..

[10] Kim D., Vazquez-Montesino L.M., Li A.A., Cholankeril G., Ahmed A. (2020). Inadequate Physical Activity and Sedentary Behavior Are Independent Predictors of Nonalcoholic Fatty Liver Disease. Hepatology.

[11] Pinto A.J., Bergouignan A., Dempsey P.C., Roschel H., Owen N., Gualano B., Dunstan D.W. (2023). Physiology of sedentary behavior. Physiol. Rev..

[12] Bianco A., Bonfiglio C., Franco I., Bagnato C.B., Verrelli N., Stabile D., Shahini E. (2025). Sedentary Behavior as a Risk Factor for Liver Fibrosis Development in Patients with Metabolic Dysfunction-Associated Steatotic Liver Disease (MASLD): A Cross-Sectional Study. J. Clin. Med..

[13] Pälve K.S., Pahkala K., Suomela E., Aatola H., Hulkkonen J., Juonala M., Lehtimäki T., Rönnemaa T., Viikari J.S.A., Kähönen M. (2017). Cardiorespiratory Fitness and Risk of Fatty Liver: The Young Finns Study. Med. Sci. Sports Exerc..

[14] Stine J.G., Soriano C., Schreibman I., Rivas G., Hummer B., Yoo E., Schmitz K., Sciamanna C. (2021). Breaking Down Barriers to Physical Activity in Patients with Nonalcoholic Fatty Liver Disease. Dig. Dis. Sci..

[15] Alabdul Razzak I., Fares A., Stine J.G., Trivedi H.D. (2025). The Role of Exercise in Steatotic Liver Diseases: An Updated Perspective. Liver Int. Off. J. Int. Assoc. Study Liver.

[16] Prince S.A., Cardilli L., Reed J.L., Saunders T.J., Kite C., Douillette K., Fournier K., Buckley J.P. (2020). A comparison of self-reported and device measured sedentary behaviour in adults: A systematic review and meta-analysis. Int. J. Behav. Nutr. Phys. Act..

[17] Sylvia L.G., Bernstein E.E., Hubbard J.L., Keating L., Anderson E.J. (2014). Practical guide to measuring physical activity. J. Acad. Nutr. Diet..

[18] Balboa-Castillo T., Muñoz S., Serón P., Andrade-Mayorga O., Lavados-Romo P., Aguilar-Farias N. (2023). Validity and reliability of the international physical activity questionnaire short form in Chilean adults. PLoS ONE.

[19] Skender S., Ose J., Chang-Claude J., Paskow M., Brühmann B., Siegel E.M., Steindorf K., Ulrich C.M. (2016). Accelerometry and physical activity questionnaires-a systematic review. BMC Public Health.

[20] Prince S.A., Adamo K.B., Hamel M.E., Hardt J., Gorber S.C., Tremblay M. (2008). A comparison of direct versus self-report measures for assessing physical activity in adults: A systematic review. Int. J. Behav. Nutr. Phys. Act..

[21] Craig C.L., Marshall A.L., Sjöström M., Bauman A.E., Booth M.L., Ainsworth B.E., Pratt M., Ekelund U., Yngve A., Sallis J.F. (2003). International physical activity questionnaire: 12-country reliability and validity. Med. Sci. Sports Exerc..

[22] Suau Q., Bianchini E., Bellier A., Chardon M., Milane T., Hansen C., Vuillerme N. (2024). Current knowledge about ActiGraph GT9X link activity monitor accuracy and validity in measuring steps and energy expenditure: A systematic review. Sensors.

[23] Migueles J.H., Cadenas-Sanchez C., Ekelund U., Delisle Nyström C., Mora-Gonzalez J., Löf M., Labayen I., Ruiz J.R., Ortega F.B. (2017). Accelerometer Data Collection and Processing Criteria to Assess Physical Activity and Other Outcomes: A Systematic Review and Practical Considerations. Sports Med..

[24] Baumann S., Groß S., Voigt L., Ullrich A., Weymar F., Schwaneberg T., Dörr M., Meyer C., John U., Ulbricht S. (2018). Pitfalls in accelerometer-based measurement of physical activity: The presence of reactivity in an adult population. Scand. J. Med. Sci. Sports.

[25] Troiano R.P., Berrigan D., Dodd K.W., Masse L.C., Tilert T., McDowell M. (2008). Physical activity in the United States measured by accelerometer. Med. Sci. Sports Exerc..

[26] Fanning J., Miller M.E., Chen S.-H., Davids C., Kershner K., Rejeski W.J. (2022). Is wrist Accelerometry suitable for threshold scoring? A comparison of hip-worn and wrist-worn ActiGraph data in low-active older adults with obesity. J. Gerontol. Ser. A.

[27] Liu F., Wanigatunga A.A., Schrack J.A. (2021). Assessment of physical activity in adults using wrist accelerometers. Epidemiol. Rev..

[28] Lu M.-J., Zhong W.-H., Liu Y.-X., Miao H.-Z., Li Y.-C., Ji M.-H. (2016). Sample size for assessing agreement between two methods of measurement by Bland—Altman method. Int. J. Biostat..

[29] Mansournia M.A., Waters R., Nazemipour M., Bland M., Altman D.G. (2021). Bland-Altman methods for comparing methods of measurement and response to criticisms. Glob. Epidemiol..

[30] Martin B.J., Altman D. (1986). Statistical methods for assessing agreement between two methods of clinical measurement. Lancet.

[31] Gerber L., Otgonsuren M., Mishra A., Escheik C., Birerdinc A., Stepanova M., Younossi Z. (2012). Non-alcoholic fatty liver disease (NAFLD) is associated with low level of physical activity: A population-based study. Aliment. Pharmacol. Ther..

[32] Watkinson C., van Sluijs E.M., Sutton S., Hardeman W., Corder K., Griffin S.J. (2010). Overestimation of physical activity level is associated with lower BMI: A cross-sectional analysis. Int. J. Behav. Nutr. Phys. Act..

[33] Ferrari G.L.d.M., Kovalskys I., Fisberg M., Gómez G., Rigotti A., Sanabria L.Y.C., García M.C.Y., Torres R.G.P., Herrera-Cuenca M., Zimberg I.Z. (2020). Comparison of self-report versus accelerometer–measured physical activity and sedentary behaviors and their association with body composition in Latin American countries. PLoS ONE.

[34] Yano S., Koohsari M.J., Shibata A., Ishii K., Mavoa S., Oka K. (2019). Assessing physical activity and sedentary behavior under Free-Living conditions: Comparison of active style pro HJA-350IT and ActiGraphTM GT3X+. Int. J. Environ. Res. Public Health.

[35] Ramalho A., Petrica J. (2023). The quiet epidemic: An overview of emerging qualitative research trends on sedentary behavior in aging populations. Healthcare.

[36] Crutzen R., Göritz A.S. (2011). Does social desirability compromise self-reports of physical activity in web-based research?. Int. J. Behav. Nutr. Phys. Act..

[37] Adams S.A., Matthews C.E., Ebbeling C.B., Moore C.G., Cunningham J.E., Fulton J., Hebert J.R. (2005). The effect of social desirability and social approval on self-reports of physical activity. Am. J. Epidemiol..

[38] Copeland J.L., Clarke J., Dogra S. (2015). Objectively measured and self-reported sedentary time in older Canadians. Prev. Med. Rep..

[39] Chastin S.F., Dontje M.L., Skelton D.A., Čukić I., Shaw R.J., Gill J., Greig C., Gale C., Deary I., Der G. (2018). Systematic comparative validation of self-report measures of sedentary time against an objective measure of postural sitting (activPAL). Int. J. Behav. Nutr. Phys. Act..

[40] Bull F.C., Al-Ansari S.S., Biddle S., Borodulin K., Buman M.P., Cardon G., Carty C., Chaput J.-P., Chastin S., Chou R. (2020). World Health Organization 2020 guidelines on physical activity and sedentary behaviour. Br. J. Sports Med..

[41] Dasso N.A. (2019). How is exercise different from physical activity? A concept analysis. Proceedings of the Nursing Forum.

[42] Keating S.E., Hackett D.A., George J., Johnson N.A. (2012). Exercise and non-alcoholic fatty liver disease: A systematic review and meta-analysis. J. Hepatol..

[43] Mambrini S.P., Grillo A., Colosimo S., Zarpellon F., Pozzi G., Furlan D., Amodeo G., Bertoli S. (2024). Diet and physical exercise as key players to tackle MASLD through improvement of insulin resistance and metabolic flexibility. Front. Nutr..

[44] Bacchi E., Negri C., Targher G., Faccioli N., Lanza M., Zoppini G., Zanolin E., Schena F., Bonora E., Moghetti P. (2013). Both resistance training and aerobic training reduce hepatic fat content in type 2 diabetic subjects with nonalcoholic fatty liver disease (the RAED2 Randomized Trial). Hepatology.

[45] Abassi W., Ouerghi N., Hammami M.B., Jebabli N., Feki M., Bouassida A., Weiss K., Knechtle B. (2025). High-intensity interval training reduces liver enzyme levels and improves MASLD-Related biomarkers in overweight/obese girls. Nutrients.

[46] Gall N., Sun R., Smuck M. (2022). A comparison of wrist-versus hip-worn ActiGraph sensors for assessing physical activity in adults: A systematic review. J. Meas. Phys. Behav..

