# Supplementary figures and images for: Beyond “Move More”: Combined Physical Activity and Sedentary Behavior Assessment in Individuals with MASLD from Southern Italy
Source: J Clin Med. 2026 Mar 11;15(6):2126. doi: 10.3390/jcm15062126 (PMC13026233; doi:10.3390/jcm15062126)

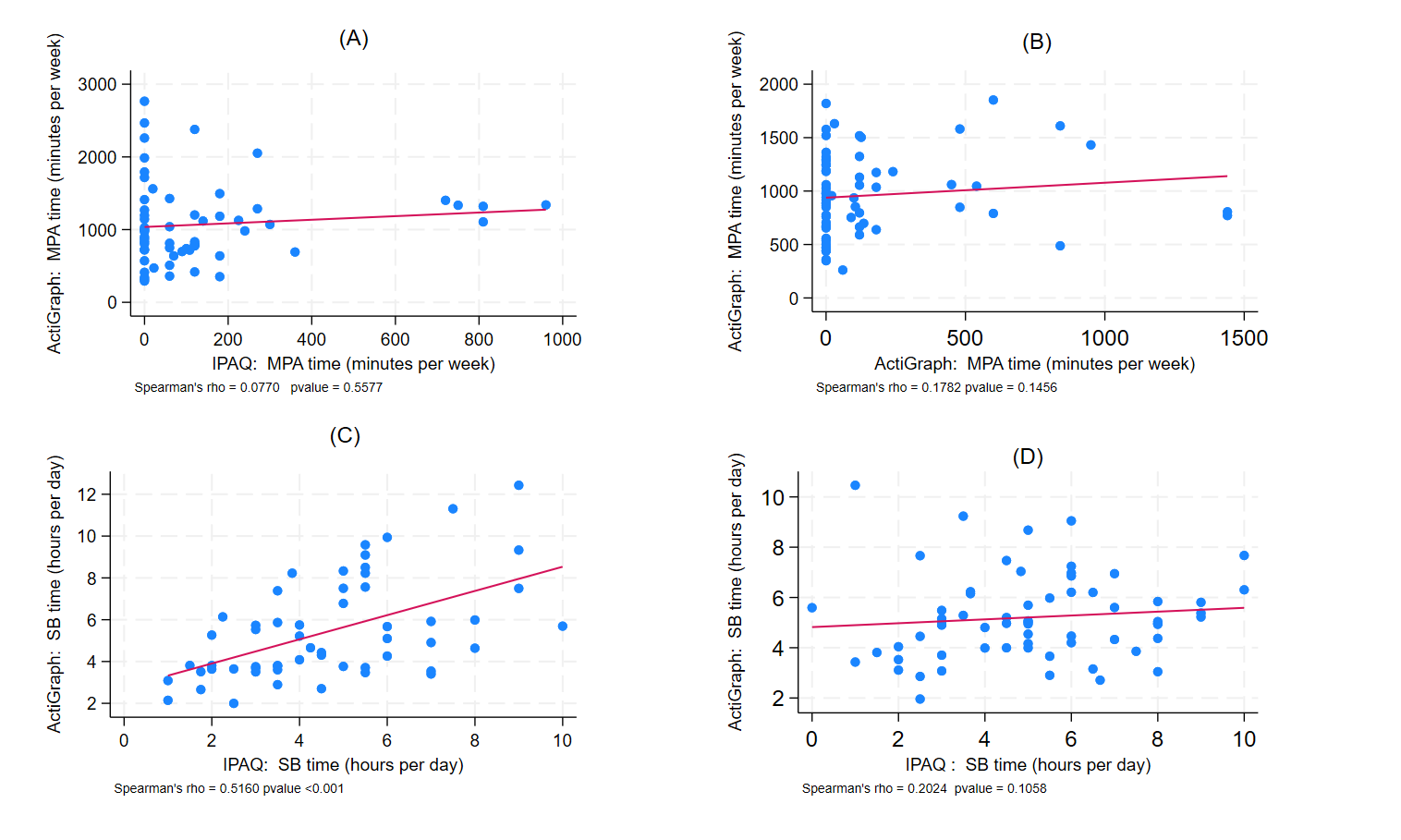

Supplement: Supplementary file 1 [file jcm-15-02126-s001.zip › Supplementary_Data.Figure S1.tif]

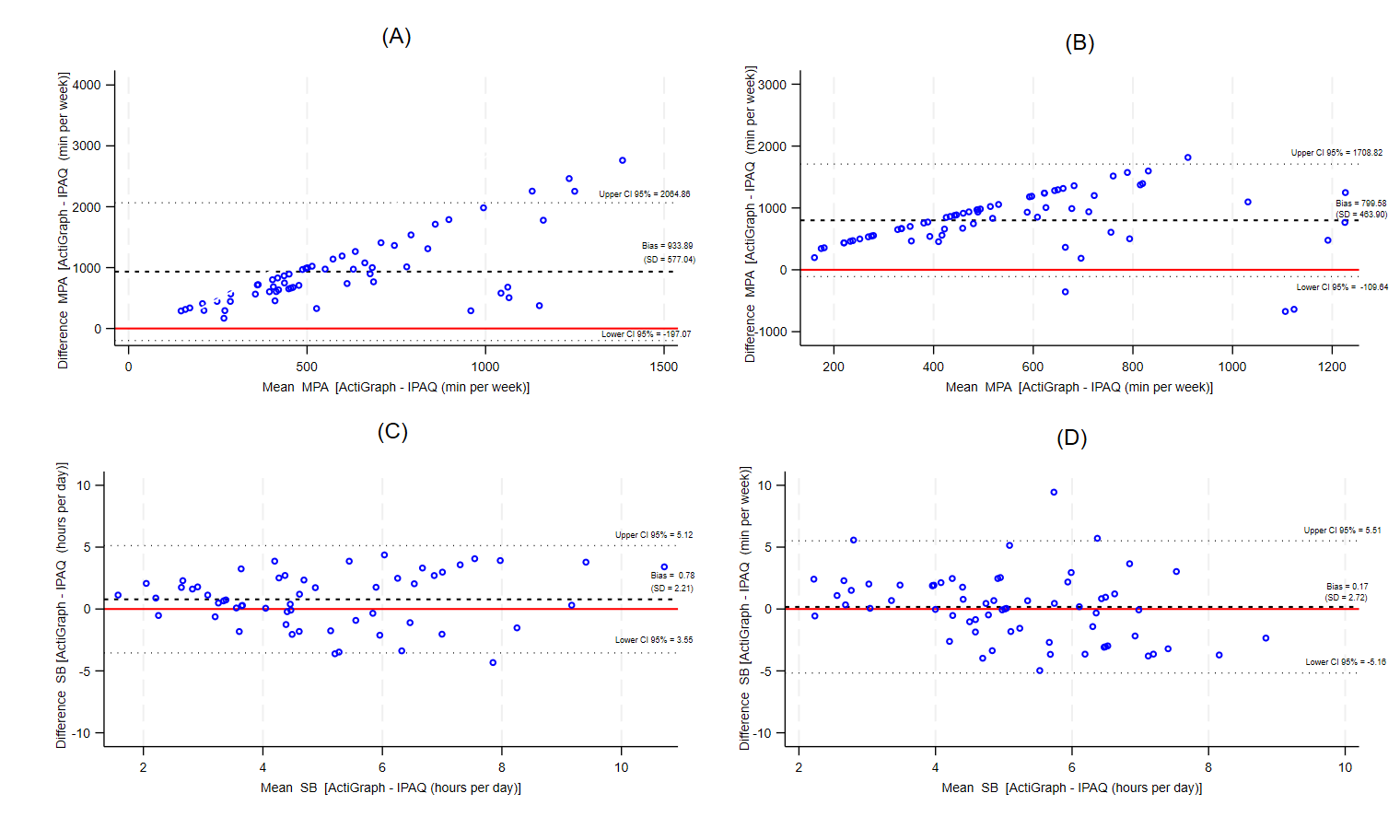

Supplement: Supplementary file 1 [file jcm-15-02126-s001.zip › Supplementary_Data.Figure S2.tif]

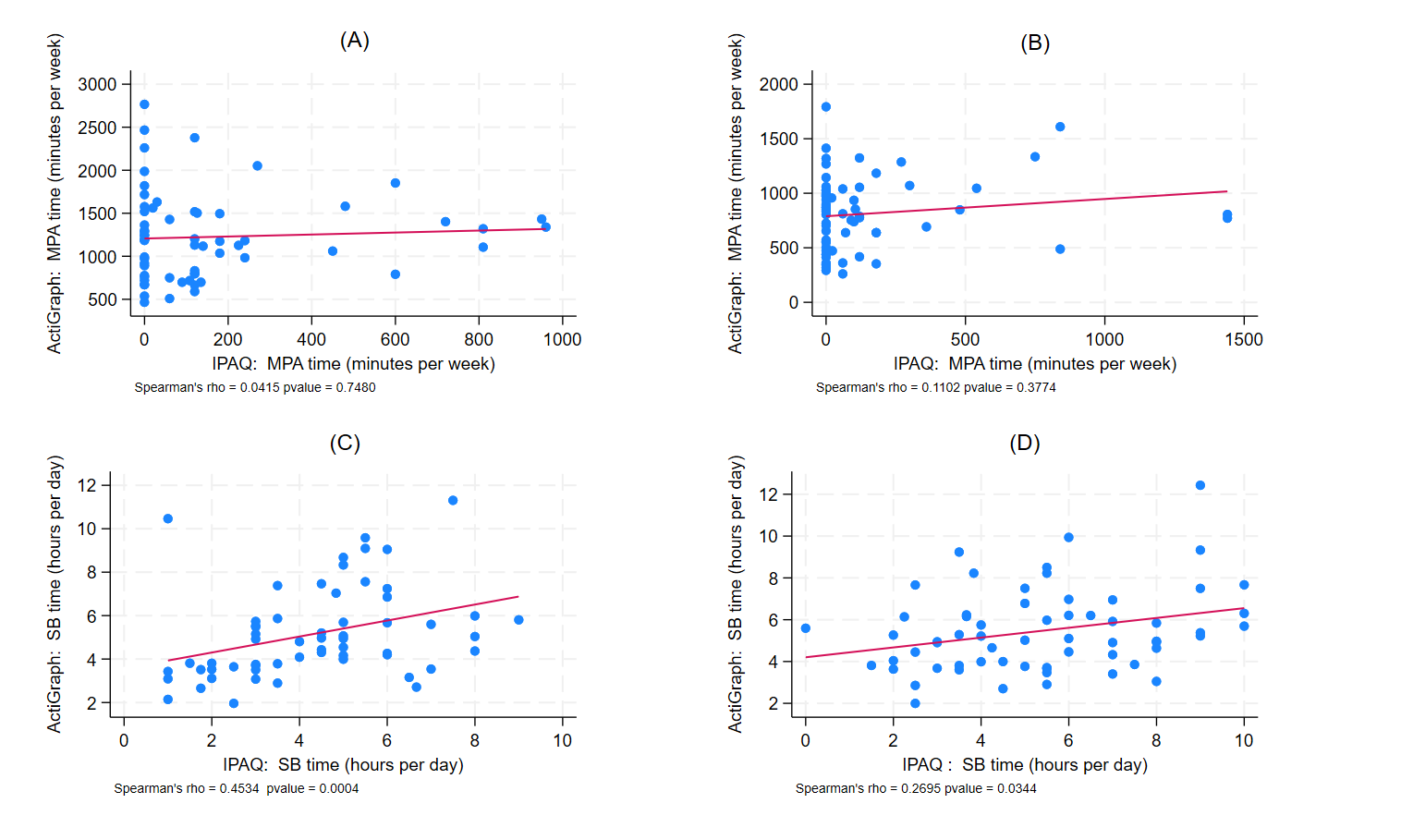

Supplement: Supplementary file 1 [file jcm-15-02126-s001.zip › Supplementary_Data.Figure S3.tif]

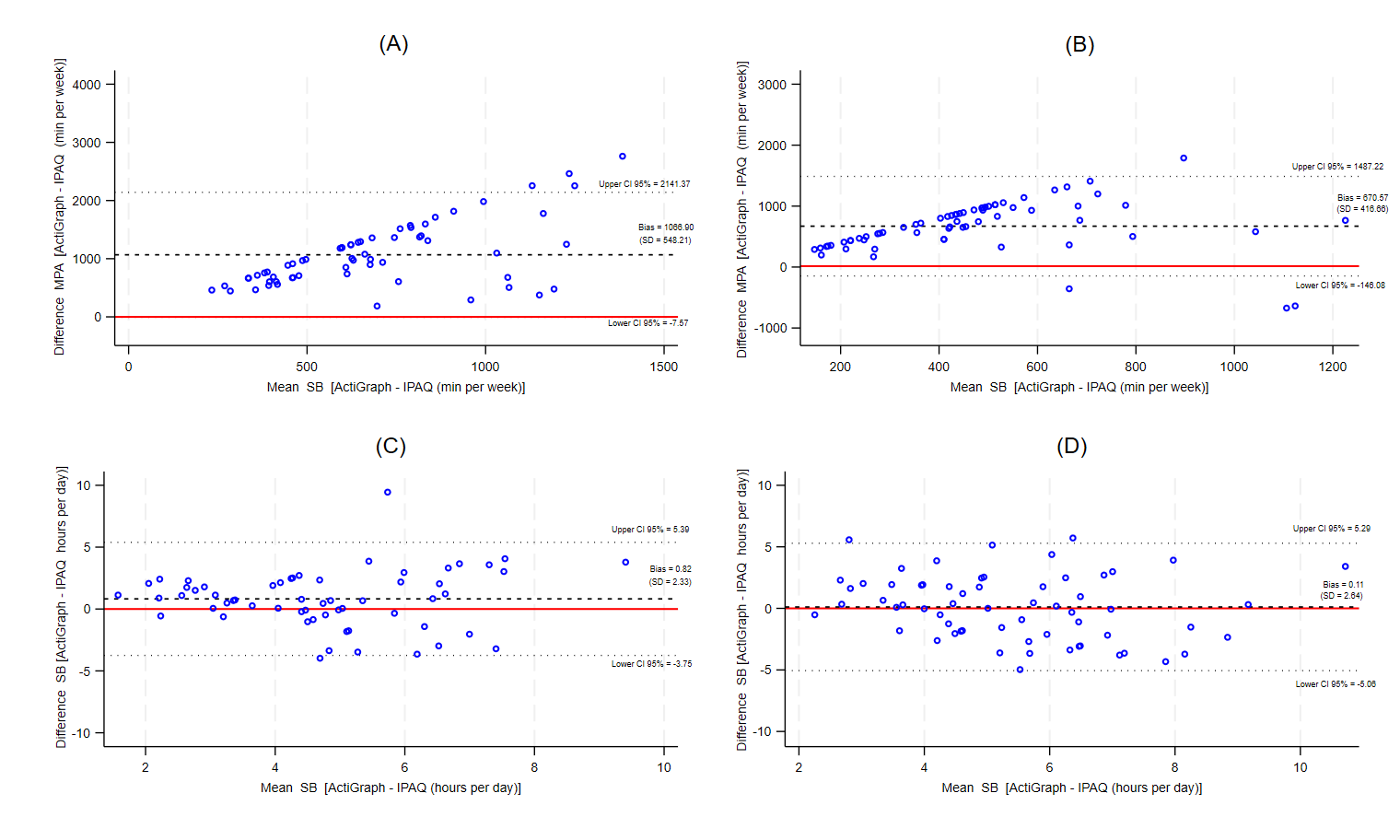

Supplement: Supplementary file 1 [file jcm-15-02126-s001.zip › Supplementary_Data.Figure S4.tif]
